# Supplementary material for: How can artificial intelligence boost firms’ exports? evidence from China
Source: PLoS One. 2023 Aug 23;18(8):e0283230. doi: 10.1371/journal.pone.0283230 (PMC10446186; doi:10.1371/journal.pone.0283230)
Supplement: Attachment — Submitted filename: Response to Reviewers.docx [file pone.0283230.s001.docx]

Responses to Reviewers

Thank you very much for the reviewers' comments concerning our manuscript entitled “How can artificial intelligence boost firms' exports? Evidence from China” (Manuscript Number PONE-D-22-32882). The comments are valuable and helpful for improving our paper. We have studied the comments carefully and made corrections according to the suggestions. A revised manuscript with the modified sections marked in red is attached as supplemental material for ease of checking.

The responses and modifications are as follows:

**Reviewer 1**

*Please provide all the data that supports these findings.*

*Very nice written introduction with clearly presented references.*

*Moreover, There are a few suggestions*

*1. Literature reviews can be more structured*

Response to comment:

Thanks for the comment and suggestion. We have adjusted the structure of this article and modified the literature review. The literature review has been removed from the introduction. The reviewer can see this in the revised manuscript.

*2. Consider the numerical optimization of Table 1,to be more visible*

Response to comment:

Thanks for the comment and suggestion. Due to the large difference between the values and units of variables, we cannot convert Table 1 into a figure. We have modified Table 1 and changed the numerical value expressed by scientific notation into decimal expression. The modified Table 1 is shown below.

**Table 1 Descriptive statistics of the variables**

| Variables | mean | Standard  deviation | maximum | minimum | samples |
| --- | --- | --- | --- | --- | --- |
| Value of export | 438403 | 14954772 | 16943636480 | 0 | 9174929 |
| TFP (OP Method, industrial value as Y, 5%) | 3.645 | 0.913 | 10.54 | -6.702 | 2860794 |
| TFP (OP Method, industrial value as Y, 10%) | 3.780 | 0.922 | 11.72 | -6.501 | 2834263 |
| TFP (OP Method, industrial value as Y, 15%) | 3.889 | 0.933 | 10.61 | -6.317 | 2788938 |
| TFP (OP Method, gross industrial output as Y, 5%) | 5.766 | 0.996 | 12.62 | -7.133 | 8332796 |
| TFP (OP Method, gross industrial output as Y, 10%) | 6.482 | 1.013 | 13.48 | -6.144 | 7858191 |
| TFP (OP Method, gross industrial outpu as Y, 15%) | 6.075 | 0.997 | 13.01 | -6.832 | 7549480 |
| TFP（LP Method） | 7.272 | 1.179 | 12.77 | -2.638 | 2860794 |
| Tariff | 6.019 | 9.905 | 1815 | 0 | 8530032 |
| assets | 308680 | 2869772.710 | 100447392 | 0 | 9174929 |
| GDP of importing countries | 2394568592 | 4005557588.007 | 16270135296 | 78900 | 9019530 |
| Capital-labor ratio | 64.81 | 613.9 | 424535 | -328.0 | 8987878 |
| Quality of export products | 0.517 | 0.179 | 1 | 0 | 8980252 |

Note: 5%, 10% and 15% in Table 1 means the capital input is calculated using the perpetual inventory method, and the capital stock was estimated at a 5%, 10% or 15% depreciation rate.

*3. The article does not yet reflect the point of innovation*

Response to comment:

Thanks for the comment. We have explained the innovation of the article in the contribution part of the introduction, and have rewritten the contribution part with major revisions. The contributions and innovation of this article are as follows:

i) Based on the existing literature on AI affecting firm productivity and employment, the productivity effect and labour substitution effect of AI affecting firm export trade behaviour are proposed. This research perspective of identifying productivity and labour substitution effects is more conducive to simplifying the complex mechanism of artificial intelligence affecting firms' exports.

ii) Matching the industrial robot data at the industry level of the International Robotics Federation with Chinese industrial enterprise data and customs data, this paper constructs the micro-data basis of the impact of AI and industrial robots on firms' exports. The database in this paper can be used as a data sample to study the impact of AI on export behaviours of Chinese enterprises.

iii) Exploring the mechanism of the impact of AI and industrial robot use on firms' export behaviour, and then answering the question of which factors lead to different trade performances of heterogeneous firms after using industrial robots, provides possible path for identifying how AI can better promote China's opening-up.

*4. Note the format of the references*

*“The authors based on Evidence from China divides the impact mechanism into productivity effect and labor substitution effect” The manuscript title,abstract and conclusion is very clear and presented well.The introduction gives a thorough description,followed by methodology adopted and discussions of the results.*

*In my opinion,the manuscript is well formatted and written well with a significant work and hence I would suggest acceptance in the present form.*

Response to comment:

Thanks for the comment and suggestion. We have checked the format of the references carefully and made modifications according to PLOS ONE guidelines for

references. The reviewer can see the modifications in the revised manuscript.

**Reviewer 2**

*By using Chinese Custom data, the Chinese Industrial Firm data and the robot data from the International Robot Federation (IRF)，the manuscript discussed the impact of industrial robots on firms' export value. Then, the manuscript conducted mechanism and heterogeneity analysis. The perspective of this research is interesting and innovative. However, the author should further elaborate the paper. As such I suggest the following suggestions.*

*1.Abstract. The authors argue that “The impact of artificial intelligence on firms' export value also varies by time period”. However, what are the different impacts? It needs to be clarified.*

Response to comment:

Thanks for the comment and suggestion. In the abstract, we argue that “the impact of artificial intelligence on firms' export value also varies by time period”, and we found two turning points: one was in 2003, before which the use of industrial robots mainly showed an inhibiting effect on firms' exports, which was gradually reflected as a driving effect thereafter; the second was in 2006, before which the impact of industrial robots on firms' exports was not significant, and after which the use of industrial robots began to significantly promote firms' exports. We have added this to the abstract of revised manuscript.

*2. Introduction. There are relatively few related discussions on the impact of AI and industrial robots on firms' export, which needs to be further strengthened to highlight the necessity of this study*.

Response to comment:

Thanks for the reminder. We have adjusted the structure of this article and removed the literature review from the introduction to make the latter clearer. The reviewer can see this in the revised manuscript.

We propose the following points to prove the necessity of this study:

i) Export transformation is urgent for China due to the gradual loss of the demographic dividend and the advantages of processing OEM.

ii) China has gradually been putting industrial robots into use in industrial production, which provides a basis for China to promote exports through artificial intelligence.

iii) The existing literature has also proved that the use of artificial intelligence and industrial robots can promote the improvement of total factor productivity, and the improvement of productivity can help promote the exports of enterprises. Thus, we believe it is necessary to verify whether and how AI and industrial robots can promote firms' exports empirically.

The above content has been included in the introduction. The reviewer can see this in the revised manuscript.

*3. The contributions. The contributions in the manuscript are like explaining the research content, rather than contributions. The author's contributions need to be further refined.*

Response to comment:

Thanks for the comment and suggestion. We have rewritten the contribution part. This part is now not only about the research content but also about the research contributions. The contributions of this article are as follows, and the reviewer can also see them in the revised manuscript.

i) Based on the existing literature on AI affecting firm productivity and employment, the productivity effect and labour substitution effect of AI affecting firm export trade behaviour are proposed. This research perspective of identifying productivity and labour substitution effects is more conducive to simplifying the complex mechanism of artificial intelligence affecting firms' exports.

ii) Matching the industrial robot data at the industry level of the International Robotics Federation with Chinese industrial enterprise data and customs data, this paper constructs the micro-data basis of the impact of AI and industrial robots on firms' exports. The database in this paper can be used as a data sample to study the impact of AI on export behaviours of Chinese enterprises.

iii) Exploring the mechanism of the impact of AI and industrial robot use on firms' export behaviour, and then answering the question of which factors lead to different trade performances of heterogeneous firms after using industrial robots, provides possible path for identifying how AI can better promote China's opening-up.

*4. On page 9, the chapter arrangement does not correspond to the article, check it.*

Response to comment:

Thanks for the reminder. We have checked the chapter arrangement outlined on page 9 and rewritten it. The reviewer can see this on page 5 in the revised manuscript.

*5. Theoretical hypotheses.*

*1) The hypothesis to be tested in the paper (such as H1, H2, H3, etc.) should be clearly pointed out. 2) The paper demonstrates the effect of time heterogeneity in the empirical analysis, but this is not shown in the theoretical hypothesis part. It is suggested to supplement.*

Response to comment:

Thanks for the suggestion.

i) We have rewritten the theoretical hypotheses part, and clearly listed the hypotheses as follows:

H1: The overall impact of AI and industrial robots on Chinese firms' exports depends on which effect dominates, the productivity effect or the substitution effect.

H2: The impact of AI and industrial robots varies significantly by industry and the export value of firms from high-tech industries benefits from the use of industrial robots.

ii) The time heterogeneity of the impact of AI on firms' exports is gradually discovered in the process of empirical research. It is not an original hypothesis based on theoretical analysis about productivity and the substitution effect. Therefore, we cannot list it in the theoretical hypotheses part.

*6. Data.*

*On page 11, “In this paper, only the data of this database from 2002 to 2013 are selected, mainly because it is difficult to estimate the total factor productivity of enterprises due to the lack of statistics of industrial value added, intermediate inputs and other indicators in the data of Chinese industrial enterprises after 2007, and there are problems of inconsistency in the selection criteria of sample enterprises and the statistical caliber of variables”.*

*The article mentioned the lack of industrial value added and there are problems of inconsistency in the selection criteria of sample enterprises and the statistical caliber of variables after 2007, but the author still chose the data from 2007 to 2013. I cannot understand the intention of the article. Besides, the data of 2002-2007 and 2002-2013 are all used in the following empirical analysis. So, I suggest you explain the sample time span here clearly.*

Response to comment:

Thanks very much for the comment and suggestion. This paragraph on data selection in the original manuscript was not clear enough. So, we have deleted this paragraph and explain the sample time span in the variable setting part to make it clearer.

We explain the sample time span as follows:

It would be ideal to estimate total factor productivity using the OP method with industrial value-added as output Y. But the statistics on industrial added value indicators are missing in the database of Chinese industrial enterprises after 2007. And the time span is also 2002-2007 using the LP method to estimate total factor productivity because the industrial value-added and intermediate input indicators are needed. Considering the time span 2002-2007 is not enough for the analysis of this article. We also estimate the total factor productivity using the OP method with gross industrial output value as output Y, and the time span for calculating is expanded to 2002-2013.

Therefore, we estimate TFP in three different ways in this article: i) the OP method with industrial value-added as output Y (2002-2007); ii) The OP method with gross industrial output value as output Y (2002-2013); iii) The LP method (2002-2007). We estimate the regression model using the different methods of estimating total factor productivity to make the results more robust. And the interpretation above is consistent with the variable setting part in the manuscript.

*7. In the article, both OP and LP methods are used to calculate TFP, but which measurement method was used in Table 2 has not been explained.*

Response to comment:

Thanks for the reminder. We have made additions to explain the measurement method used in Table 2. In the benchmark regression, we use the OP method to measure TFP with industrial value-added as the output variable, and the capital stock is estimated using the perpetual inventory method using 10% depreciation rates. The reviewer can see these changes on page 16 in the revised manuscript.

*8. When measuring enterprise’s TFP with LP, it is suggested that capital input, labor input and intermediate input variables should be supplemented.*

Response to comment:

Thanks for the suggestion. We use both the OP and LP methods to calculate TFP. When the OP method is used, we calculate the capital input using the perpetual inventory method and take the number of employees as labour input. The data on total intermediate input can be found in the database of Chinese industrial enterprises before 2008, so we can estimate TFP using the LP method in the period 2002-2007. The above content has been added to the revised manuscript (Page14-15).

*9. All empirical regression tables are not standardized and lack of annotations. The meaning of the value in brackets and the significance level of the asterisk is not explained, although it does not affect the understanding of the empirical results.*

Response to comment:

Thanks for the suggestion. We have provided an additional explanation about the significance level under Table 2 (on page 17 in the revised manuscript), and the value in brackets is the standard error.

*10. On Page 18, the interpretation of the results in Table 3 does not correspond to the contents of the table, the author needs to check it carefully.*

Response to comment:

Thanks for the reminder. We have carefully checked the interpretation of the results in Table 3 against the content of this table and confirm that it is consistent with the interpretation.

*11.Table 3.*

*1) The results in columns (3) and (4) in Table 3 are not significant, and the coefficient in column (5) is significantly positive, which is inconsistent with the benchmark analysis. Can it indicate that the results are robust?*

*2) It is suggested to add robustness testing methods to enhance the reliability of the results. For example, change the estimation sample, change the variable measurement method, etc.*

Response to comment:

Thanks for the comment and suggestion. We see that made some mistakes in this paragraph. The paragraph before Table 3 has been modified as follows (the reviewer can also see this on page 19 in the revised manuscript):

“In Table 3, the first two columns adopt the OP method and estimate total factor productivity using industrial value-added as the output variable. Since the industrial value-added data provided in the industrial enterprise data are only from before 2008, the first two columns of the regression also span the period 2002-2007, and the difference between the first two columns is that the capital stock is estimated using the perpetual inventory method using 5% and 15% depreciation rates, respectively. Columns (3)-(5) use the OP method and estimate total factor productivity using gross industrial output (GIP) as the output variable, with data spanning 2002-2013, and columns (3), (4), and (5) use 5%, 10%, and 15% depreciation rates, respectively, to calculate the capital stock. The last column uses the LP method and estimates total factor productivity, which still spans the period 2002-2007.”

So, columns (3)-(5) use the OP method and estimate total factor productivity using gross industrial output (GIP) as the output variable, with data spanning 2002-2013, and columns (3), (4), and (5) use 5%, 10%, and 15% depreciation rates, respectively, while the first two columns adopt the OP method and estimate total factor productivity using industrial value-added as the output variable. The last column uses the LP method to estimate total factor productivity. So, the time span of the first two columns and the last column is 2002-2007.

We find that industrial robot density is significantly negatively correlated with firms' exports when TFP is estimated by the OP method using industrial value-added as an output variable or by the LP method (both with data spanning 2002-2007), while the coefficient of the industrial robot density variable is positive when TFP is estimated using gross industrial output value as an output variable (with data spanning 2002-2013). Considering the time span of the benchmark regression is also 2002-2007 and the density of industrial robots is also significantly negatively correlated with the export value, we can see there may be significant temporal differences in the effects on the export behaviour of micro-enterprises. So, the results of the regression in Table 3 are consistent with the results of the benchmark regression. The coefficient of the density of industrial robots is significantly negative with data spanning 2002-2007, while the coefficient is positive with data spanning 2002-2013. Therefore, we estimate the effect of AI and industrial robot on firms' exports with different time intervals in the following.

Thanks for the suggestion. To ensure the robustness of the estimation results, we actually change the variable measurement method (estimating TFP both by OP and LP method).

*12. On page 20, “2013 data are positively correlated at a significance level of 10%, which again reflects that the impact of AI on the export behavior of micro-enterprises in China has some temporal differences”. I don't think this explanation is reasonable. The results are based on the sample from 2002 to 2013, not 2013 data. The author should give reasonable reasons.*

Response to comment:

Thanks for the comment and suggestion. As the reviewer points out, the results are based on the sample from 2002 to 2013, not 2013 data. The statement is inaccurate in that paragraph on page 20, so we have made some modifications. The reviewer can see them on page 22 in the revised manuscript.

As can be seen from Table 4, the main difference between high-tech and non-high-tech industries is reflected in the effect of AI and industrial robots on firms' exports. The coefficient of robot density is significantly positive for high-tech industries. While a significant negative correlation between firms' exports and industrial robot density for non-high-tech firms is shown for the time span 2002-2007, the coefficient of robot density is positive for non-high-tech firms based on the sample from 2002 to 2013. The regression results vary by time period for non-high-tech firms, which again reflects that the impact of AI on the export behaviour of micro-enterprises in China has some temporal differences. And we verify this effect in the following.

*13.IV regression. In Table 8 and Table 9, the coefficients of columns (1) and (3) are negative, but not significant, which means that the benchmark regression results are not robust after considering the endogenous problem, and the reliability of the conclusion is worrying.*

Response to comment:

Thanks for the comment and suggestion. The main conclusion of this paper is that the effect of artificial intelligence and industrial robots on Chinese firms' export value is generally negative, but the export value of firms from high-tech industries benefits from the use of industrial robots.

i) The results of IV Regression (Table 8)

The first three columns of Table 8 report the results of instrumental variable estimation for the all-industry sample, and the use of industrial robots in the all-industry sample is negatively correlated with firms' exports, except for column (2) (this is consistent with the results of the benchmark regression). The latter three columns report the results of estimating instrumental variables for high-technology industries, and the use of industrial robots is positively correlated with firms' exports in high-tech industries. It is more important to verify the positive impact of industrial robots on firms' exports for high-tech industries, than to confirm the negative impact on firms' exports for the all-industry sample. So, we believe the results of IV regression are still reliable.

ii) The results of the regression with robot density one year lagged (Table 9)

In order to make the estimation results more robust and reliable, we also re-estimate the regression with robot density one year lagged. We use robot density one year lagged as the IV and re-estimate the effect of AI and industrial robots on firms' exports using the GMM method. The results are reported in the new Table 9. The reviewer can see this in the revised manuscript. The modified Table 9 is also presented below.

**Table 9 Regression results with robot density one year lagged**

|  | All-industry sample | | | | High-tech industries | | |
| --- | --- | --- | --- | --- | --- | --- | --- |
|  | (1) | (2) | (3) | (4) | | (5) | (6) |
| Variable | op10 | op10_1 | lp | op10 | | op10_1 | lp |
| Robot density | 0.051*** | 0.078*** | 0.051*** | 0.048*** | | 0.006* | 0.050*** |
|  | (20.096) | (49.678) | (20.395) | (8.291) | | (1.662) | (8.673) |
| TFP | 0.121*** | 0.460*** | 0.370*** | -0.075*** | | 0.011 | -0.098* |
|  | (8.701) | (30.340) | (12.818) | (-2.962) | | (0.393) | (-1.744) |
| Tariff | 1.102*** | 1.043*** | 1.089*** | 0.405*** | | 0.603*** | 0.384*** |
|  | (23.544) | (39.024) | (23.456) | (3.696) | | (9.804) | (3.525) |
| Firm Size | 0.193*** | 0.129*** | 0.170*** | 0.198*** | | 0.131*** | 0.202*** |
|  | (77.497) | (95.796) | (54.731) | (43.829) | | (54.553) | (33.432) |
| GDP | 0.241*** | 0.243*** | 0.241*** | 0.212*** | | 0.212*** | 0.211*** |
|  | (104.084) | (194.027) | (104.690) | (47.247) | | (89.162) | (47.364) |
| Year FE | Yes | Yes | Yes | Yes | | Yes | Yes |
| Importing country FE | Yes | Yes | Yes | Yes | | Yes | Yes |
| Product FE | Yes | Yes | Yes | Yes | | Yes | Yes |
| Industry FE | Yes | Yes | Yes | Yes | | Yes | Yes |
| Firm FE | Yes | Yes | Yes | Yes | | Yes | Yes |
| Samples | 474385 | 1881418 | 481222 | 142373 | | 605822 | 144065 |
| *R^2^* | 0.047 | 0.041 | 0.047 | 0.068 | | 0.065 | 0.067 |
| Adjusted *R^2^* | 0.047 | 0.041 | 0.047 | 0.068 | | 0.065 | 0.067 |
| *F*-value | 2320.381 | 8024.429 | 2344.391 | 886.427 | | 3394.725 | 879.564 |

*14. Mechanism test. This paper verifies the factors affecting the productivity effect and the substitution effect through interaction term, but single variables also need to be added. That is, in Table 12 industrial robot density and capital-labor ratio should add in the regression model. And, in Table 13 robot density and export products quality should add in the model. The author needs to re-estimate the results in Table 12 and Table 13.*

Response to comment:

Thanks for the comment and suggestion. As the reviewer suggested, if we want to verify the moderating effect of the capital-labour ratio and export product quality on the use of industrial robots on firms' exports by introducing the interaction term, single variables also need to be added. So, we add robot density and capital-labour ratio in Table 12, and add robot density and export product quality in Table 13. Based on that, we have rewritten the regression results analysis. The reviewer can see this in the revised manuscript. The modified Table 12 and Table 13 are also shown below.

**Table12 The Regression results with interaction term between industrial robot density and firm capital-labour ratio**

|  | All-industry sample | | | High-tech industries | | |
| --- | --- | --- | --- | --- | --- | --- |
|  | (1) | (2) | (3) | (4) | (5) | (6) |
| Variable | *op5_1* | *op10_1* | *op15_1* | *op5_1* | *op10_1* | *op15_1* |
| TFP | 0.464*** | 0.591*** | 0.422*** | 0.529*** | 0.624*** | 0.502*** |
|  | (35.625) | (37.697) | (29.097) | (20.788) | (20.389) | (18.183) |
| Tariff | -0.102*** | -0.113*** | -0.113*** | -0.026 | -0.029 | -0.023 |
|  | (-5.590) | (-6.029) | (-5.902) | (-0.639) | (-0.685) | (-0.538) |
| Robot density | 0.005 | 0.006 | 0.011 | 0.159*** | 0.151*** | 0.137*** |
|  | (0.672) | (0.909) | (1.482) | (8.221) | (7.629) | (6.776) |
| KL | 0.010*** | 0.012*** | 0.010*** | 0.012*** | 0.014*** | 0.019*** |
|  | (6.582) | (7.740) | (6.193) | (3.975) | (4.745) | (6.268) |
| Interaction term | 0.002*** | 0.001*** | 0.001*** | 0.001 | 0.001 | -0.000 |
|  | (4.646) | (2.865) | (2.747) | (0.790) | (0.620) | (-0.133) |
| Firm Size | 0.079*** | 0.081*** | 0.090*** | 0.090*** | 0.089*** | 0.101*** |
|  | (25.982) | (24.963) | (27.111) | (14.991) | (14.079) | (15.376) |
| GDP | 0.018** | 0.025*** | 0.029*** | 0.137*** | 0.139*** | 0.138*** |
|  | (2.015) | (2.718) | (3.111) | (8.206) | (8.145) | (7.975) |
| Year FE | Yes | Yes | Yes | Yes | Yes | Yes |
| Importing country FE | Yes | Yes | Yes | Yes | Yes | Yes |
| Product FE | Yes | Yes | Yes | Yes | Yes | Yes |
| Industry FE | Yes | Yes | Yes | Yes | Yes | Yes |
| Firm FE | Yes | Yes | Yes | Yes | Yes | Yes |
| Samples | 6687613 | 6304194 | 6051295 | 2159060 | 2043309 | 1965237 |
| *R^2^* | 0.333 | 0.334 | 0.335 | 0.363 | 0.364 | 0.364 |
| Adjusted *R^2^* | 0.321 | 0.322 | 0.322 | 0.351 | 0.351 | 0.350 |
| *F*-value | 348.042 | 369.277 | 276.677 | 129.796 | 123.267 | 110.041 |

**Table13 The Regression results with interaction term between industrial robot density and export products quality**

|  | All-industry sample | | | High-tech industries | | |
| --- | --- | --- | --- | --- | --- | --- |
|  | (1) | (2) | (3) | (4) | (5) | (6) |
| Variable | *op5_1* | *op10_1* | *op15_1* | *op5_1* | *op10_1* | *op15_1* |
| TFP | 0.211*** | 0.267*** | 0.156*** | 0.254*** | 0.297*** | 0.173*** |
|  | (31.504) | (32.026) | (20.620) | (17.341) | (16.381) | (10.762) |
| Tariff | -0.149*** | -0.154*** | -0.155*** | -0.361*** | -0.360*** | -0.351*** |
|  | (-13.571) | (-13.595) | (-13.430) | (-13.346) | (-12.952) | (-12.399) |
| Robot Density | -0.070*** | -0.070*** | -0.067*** | 0.236*** | 0.233*** | 0.225*** |
|  | (-17.649) | (-17.095) | (-15.926) | (19.596) | (18.794) | (17.791) |
| Product Quality | 12.585*** | 12.576*** | 12.561*** | 11.950*** | 11.953*** | 11.951*** |
|  | (2691.757) | (2603.928) | (2541.376) | (1312.376) | (1279.898) | (1256.088) |
| Interaction term | 0.014*** | 0.019*** | 0.021*** | 0.378*** | 0.386*** | 0.381*** |
|  | (8.144) | (10.793) | (11.515) | (86.120) | (86.020) | (83.254) |
| Firm Size | 0.020*** | 0.022*** | 0.026*** | 0.021*** | 0.019*** | 0.025*** |
|  | (10.998) | (11.445) | (13.381) | (5.461) | (4.625) | (5.942) |
| GDP | -0.106*** | -0.104*** | -0.101*** | -0.088*** | -0.087*** | -0.083*** |
|  | (-18.791) | (-18.106) | (-17.284) | (-7.897) | (-7.673) | (-7.242) |
| Year FE | Yes | Yes | Yes | Yes | Yes | Yes |
| Importing country FE | Yes | Yes | Yes | Yes | Yes | Yes |
| Product FE | Yes | Yes | Yes | Yes | Yes | Yes |
| Industry FE | Yes | Yes | Yes | Yes | Yes | Yes |
| Firm FE | Yes | Yes | Yes | Yes | Yes | Yes |
| Samples | 6641530 | 6261133 | 6010608 | 2129137 | 2015104 | 1938623 |
| *R^2^* | 0.747 | 0.747 | 0.747 | 0.718 | 0.718 | 0.718 |
| Adjusted *R^2^* | 0.743 | 0.742 | 0.742 | 0.712 | 0.712 | 0.712 |
| *F*-value | 1.1e+06 | 1.0e+06 | 9.7e+05 | 2.6e+05 | 2.5e+05 | 2.4e+05 |

References:

[1]Brambor T, Clark W R, Golder M. Understanding Interaction Models: Improving Empirical Analyses[J]. Political Analysis, 2006, 14(1):63-82.

[2]Hainmueller J, Mummolo J, Xu Y. How Much Should We Trust Estimates from Multiplicative Interaction Models? Simple Tools to Improve Empirical Practice[J]. SSRN working paper, 2016.

*15. At last, there are a lot of expression and typo errors, please revise and check the whole manuscript carefully. It is suggested to polish the language of the article.*

Response to comment:

Thanks for comment and suggestion. We have polished the language of the article and checked the manuscript carefully.

**Reviewer 3**

*Reconsideration after major revision.*

*The study explores the impact of artificial intelligence and industrial robots on firms' export behavior and divides the impact mechanism into productivity effect and labor substitution effect.*

*This paper examines the effect of industrial robots on firms' export value by using Chinese Custom data, the Chinese Industrial Firm data and the robot data from the International Robot Federation (IRF).*

*However, the paper is not yet suitable for publication in "PLOS ONE" and requires major revisions.*

*1. The contributions should be strengthened.*

Response to comment:

Thanks for the comment and suggestion. We have rewritten the contribution part. This part is not only about the research content but also about the research contributions. The contributions of this article are as follows (the reviewer can see them in the revised manuscript):

i) Based on the existing literature on AI affecting firm productivity and employment, the productivity effect and labour substitution effect of AI affecting firm export trade behaviour are proposed. This research perspective of identifying productivity and labour substitution effects is more conducive to simplifying the complex mechanism of artificial intelligence affecting firms' exports.

ii) Matching the industrial robot data at the industry level of the International Robotics Federation with Chinese industrial enterprise data and customs data, this paper constructs the micro-data basis of the impact of AI and industrial robots on firms' exports. The database in this paper can be used as a data sample to study the impact of AI on export behaviours of Chinese enterprises.

iii) Exploring the mechanism of the impact of AI and industrial robot use on firms' export behaviour, and then answering the question of which factors lead to different trade performances of heterogeneous firms after using industrial robots, provides possible path for identifying how AI can better promote China's opening-up.

*2. The models used in the paper are not visualized, a figure is recommended.*

*A remark and discussion on the orientation of the model is necessary.*

Response to comment:

Thanks for the suggestion. We have provided a figure to illustrate the theoretical hypotheses. The model in this article has been visualized. Figure 2 is shown below and the reviewer can see it in the revised manuscript.

Fig 2 Theoretical Hypotheses

The main orientation of the theoretical model in this article is the existing literature. We have explained it in the theoretical hypotheses section.

We propose the productivity effect based on Melitz (2003), Graetz and Michaels (2018), Kromann et al. (2020) and Ballestar et al. (2020). Graetz and Michaels (2018), Kromann et al. (2020) and Ballestar et al. (2020) prove that the use of industrial robots can promote total factor productivity by replacing low-skilled labour. Melitz (2003) stated that higher productivity firms tend to export products. Zhang and Wang (2020) also verified that the export value of enterprises is positively correlated with their productivity levels. Based on the literature, this paper proposes that the productivity effect of AI affects the exports of micro-enterprises. The use of AI and industrial robots can promote firms' exports by increasing the total factor productivity. According to the productivity effect, the use of AI is positively related to the export value of enterprises.

We propose the substitution effect based on a series of studies by Acemoglu as well as Restrepo (2017a, 2017b, 2018) and Alonso et al. (2020). The work of Acemoglu and Restrepo (2017a, 2017b, 2018) shows that the substitution of labour by industrial robots is mainly in the form of the substitution of low-skilled labour. For high-technology industries, the majority of the workforce is highly skilled, so the increase in robot use promotes product exports; whereas for non-high-technology industries, low-skilled labour is more likely to be replaced by industrial robots. Alonso et al. (2020) showed that the substitution of low-skilled labour by industrial robots will discourage firms from exporting. Therefore, we assume that for high-technology industries, the substitution effect of AI and industrial robots is reflected in a positive relationship between industrial robot density and firms' exports, while for non-high-technology industries, the substitution effect is reflected in a negative relationship between the two.

Based on the literature, we assume that for high-tech industries, both the productivity effect and the substitution effect are positive, while for non-high-tech industries, the overall effect depends on which one is superior, the substitution effect or the productivity effect.

References:

[1]Melitz MJ, The Impact of Trade on Intra-Industry Reallocations and Aggregate Industry Productivity. Econometrica. 2003; 71(6):1695-1725.

[2]Graetz G, Michaels G. Robots at Work. Review of Economics and Statistics. 2018; 100(5): 753-768.

[3]Kromann L, Malchow-Møller N, Skaksen JR, Sørensen A, Automation and productivity-a cross-country, cross-industry comparison. Industrial and Corporate Change. 2020; 29(2): 265–287.

[4]Ballestar MT, Diaz-Chao A, Sainz J, et al. Knowledge, robots and productivity in SMEs: Explaining the second digital wave. Journal of Business Research. 2020; 108(1):119-131.

[5]Acemoglu D. Restrepo P. The Race between Machine and Man: Implications of Technology for Growth, Factor Shares and Employment. American Economic Review. 2018; 108(6): 1488-1542.

[6]Acemoglu D, Restrepo P. Robots and jobs: Evidence from US labor markets. Journal of Political Economy. 2020; 128(6): 2188-2244.

[7]Acemoglu D, Lelarge C, Restrepo P. Competing with Robots: Firm-Level Evidence from France. CEPR Discussion Papers. 2020.

[8]Alonso C, Berg A, Kothari S, Papageorgiou C, Rehman S, 2020. Will the AI Revolution Cause a Great Divergence?. Journal of monetary economics. 2022; 127(4): 18-37.

*3. Variable selection:*

*The reasons of variables selection are strongly proposed to present.*

Response to comment:

Thanks for the suggestion. We have presented the reasons of variable selection (especially the control variables) in the manuscript.

Besides the dependent variable (firms' exports) and independent variable (robot density), we also introduce total factor productivity, tariffs and GDP of importing countries, and firm size as control variables.

We explain the reasons for the control variable selection as follows:

i) Total factor productivity. New-new trade theory suggests that firms' export behaviour is a self-selection of high-productivity firms (Melitz, 2003). High-productivity firms will prefer to export, based on which this paper adds total factor productivity as a control variable to the empirical model.

ii) GDP of importing countries. GDP of importing countries is included to reflect the impact of a demand-side factor on firms' export behaviour. It is assumed that firms tend to export more to larger destination countries.

iii) Tariff of importing countries. According to the classical trade theory and the gravity model, tariff is an important factor affecting export value.

iv) Firm size. Based on Fontagné et al. (2015) and other papers, the size of the enterprise affects its export value, and larger enterprises tend to export more. Thus, this paper uses total firm assets as an indicator of firm size to measure a supply-side factor which affects export value and uses it as a control variable for firm characteristics.

We also introduce capital-labour ratio and export product quality to construct the interaction term. The reason we introduce capital-labour ratio to construct the interaction term is presented in a simple model based on Korinek and Stiglitz (2021) from Part 4.6.2. We introduce export product quality to construct the interaction term because we assume high-value-added products require more high-skilled labour to produce, so industrial robots will mainly reflect the productivity effect, but less the substitution effect of low-skilled labour, so the use of industrial robots will eventually lead to an increase in firms' exports.

References:

[1]Melitz MJ, The Impact of Trade on Intra-Industry Reallocations and Aggregate Industry Productivity. Econometrica. 2003; 71(6):1695-1725.

[2]Fontagné L, Orefice G, Piermartini R, Rocha N. Product Standards and Margins of Trade: Firm-Level Evidence. Journal of International Economics. 2015; 97(1): 29-44.

[3]Korinek A, Stiglitz JE. Artificial Intelligence, Globalization, and Strategies for Economic Development. NBER Working Paper. 2021.

*4. It is unclear how the results presented can be used to support the decision makers.*

*There are many SERIOUS spelling and style problems in the paper, such as:*

*(1)The initial letters of the key words should be capitalized.*

*(2) For Chinese Customs, the ‘s’ is necessary.*

*(3) Reference style.*

*(4) Numbering.*

*(5) Brynjolfsson and McAfee (2014) referred to AI and the digital revolution as the second machine revolution, arguing that it is as important as the industrial revolution in driving development [4].*

*Brynjolfsson E, Mcafee A . The Second Machine Age[J]. Nz Business, 2014, 14(11):1895-1896.*

Response to comment:

Thanks for the comment and suggestion. We have carefully checked the spelling and numbering problems in this paper, capitalized the initial letters of the key words and changed all the instances of "custom" into "customs".

We have checked the reference style carefully and made corresponding modifications.

We have also had this manuscript proofread. The reviewer can see this in the revised manuscript.
