# Supplementary material for: How can artificial intelligence boost firms’ exports? evidence from China
Source: PLoS One. 2023 Aug 23;18(8):e0283230. doi: 10.1371/journal.pone.0283230 (PMC10446186; doi:10.1371/journal.pone.0283230)
Supplement: Attachment — Submitted filename: Response to Reviewers.docx [file pone.0283230.s002.docx]

Responses to Reviewers

Thanks very much for the reviewers’ comments concerning our manuscript entitled “How can artificial intelligence boost firms' exports? Evidence from China” (Manuscript Number PONE-D-22-32882). The comments are very helpful for improving our paper. A revised manuscript with the modified sections marked in red is attached as supplemental material for ease of checking.

The responses and modifications are as follows:

**Reviewer 2**

*The author made detailed modifications to my comments. I am very satisfied with these modifications.*

Response to comment:

Thanks for the comment. The reviewer’s suggestions are very helpful for us to improve the paper.

**Reviewer 3**

*The language of the paper still has problems of expression and grammatical errors. The author is requested to revise the language carefully to make the language correct, clear and in line with academic norms.*

Response to comment:

Thanks for the comment and suggestion. We have carefully checked the problems of expression and grammatical errors in this paper. We have also polished the language of the article again and revised the manuscript carefully. The reviewer can see this in the revised manuscript.
